# Household Food Insecurity and SNAP Dynamics: Implications for Cognitive Health and Well-Being in Later Life.

**DOI:** 10.1093/geroni/igaf122.1411

**Published:** 2025-12-31

**Authors:** Zuo Dongmei, Colleen Heflin

**Affiliations:** Emory University, Atlanta, Georgia, United States; Syracuse University, Maxwell School of Citizenship and Public Affairs, Syracuse, New York, United States

## Abstract

This research examines how food insecurity (FI) and public assistance exposure are associated with later-life cognitive health and economic well-being from a life course perspective. Using Health and Retirement Study (1995–2020) data, we find that midlife FI can be persistent, with longer exposure during one’s fifties significantly linked to lower cognitive functioning after age 60. These results underscore how early economic disadvantage can have enduring health consequences and highlight the potential protective role of SNAP participation against cognitive decline. In a complementary analysis, we find that as individuals transition into cognitive impairment, they face substantial barriers to enrolling in SNAP, which we attribute to potential difficulties with administrative burdens. Our results suggest that the administrative burden inherent in SNAP enrollment may contribute to lower take-up rates among cognitively impaired seniors. The efforts to simplify eligibility and recertification processes may help improve access to food assistance for this vulnerable group. Extending the inquiry into the familial domain, we assess how intergenerational and intragenerational dynamics influence SNAP usage. Evidence from the Panel Study of Income Dynamics (1975–2019) shows that early exposure to SNAP during adolescence, along with parental and sibling participation, is significantly associated with an individual’s own increased likelihood of enrollment. This indicates that family networks play a pivotal role in mitigating stigma and administrative burdens associated with public assistance, while also buffering against food insecurity—particularly among disadvantaged populations. Encouraging whole-family outreach and educational programs may strengthen benefit communication and access, particularly in communities where familial influence is especially pronounced.

